# Resisting the politics of the pandemic and racism to foster humanity

**DOI:** 10.1177/1473325020973313

**Published:** 2021-03

**Authors:** Eunjung Lee, Marjorie Johnstone

**Affiliations:** Factor-Inwentash Faculty of Social Work, University of Toronto, Toronto, ON, Canada; School of Social Work, Dalhousie University, Halifax, NS, Canada

**Keywords:** Politics of the pandemic, racism, COVID-19, anti-immigration, research as resistance, and social justice

## Abstract

Following the World Health Organization (WHO) declaration of COVID-19 as a pandemic in March 2020, a state of emergency was announced in many countries. This has had significant impacts on individuals, communities, and various systems-as-whole locally, nationally and globally. Among the various impacts the pandemic has on people, we would like to invite social workers who deeply care about social justice and equity to pause and reflect on how some populations are unjustly subject to pandemic related stigma and racism; how racist politics play out to maintain extreme nationalism and exclusion; and how we can resist these politics of the pandemic to foster humanity and equity.

After all, it really is all of humanity that is under threat during a pandemic.

Margaret Chan, Former Director-General of WHO

## Pandemic and racism in the globe

Despite being a novel virus, COVID-19 echoes other pandemics from the Spanish Flu to the Middle Eastern Respiratory Syndrome (MERS), with the appearance of social stigma related to geographical area, ethnicity and race, all of which are *prototypes of xenoracism* (Maunder et al., 2008; Serhan and McLaughlin, 2020). COVID-19, mislabelled as a foreign virus, has had ramifications for people of Asian descent, as evidenced in COVID related hate crimes globally. In the United States, federal law enforcement has warned of increased hate crimes against Asian Americans and has listed incidents from Los Angeles to New York to Texas (Margolin, 2020). On March 14, an extreme case was in Midland, Texas where three Asian American family members, including a 2-year-old and a 6-year-old, were stabbed since the suspect thought the family was Chinese infecting people with the coronavirus. In Vancouver, Canada, on March 13, a 92-year-old Asian man with dementia, was yelled at with comments about COVID-19 and then shoved, so he fell and hit his head. Vancouver police reported that hate crimes against people of East Asian descent in Vancouver doubled in April 2020 (Hager, 2020). In Melbourne, Australia, on April 15, two Chinese international students were brutally kicked and yelled at with ‘Go back to China’ (Sakkal, 2020). In the United Kingdom (UK), people are requesting not to be treated by Asian doctors and nurses despite the fact that health professionals are most needed during this health crisis (Qasim, 2020). In May 2020, *Human Rights Watch* reported numerous incidents of xenophobic attacks and harassment of people of Asian descent in Australia, Brazil, Ethiopia, France, Kenya, India, Italy, Russia, South Africa, Sri Lanka, and UK. Carol Liao (2020), a law professor at the University of British Columbia, raised concerns about how some people are ‘treating COVID-19 as a licence to exhibit hate, only emphasizing the long history of racism.’ In this reflexive essay, we thus explore pervasive narratives around the pandemic and underlying ever-present xenoracism, white supremacy, and far-right nationalism in the era of COVID-19.

## Politics of the pandemic and racism

While originating in China and then spreading to other Asian countries before the West, the coronavirus is accused of being located in Chinese and Asian bodies, reifying racist anti-Chinese and anti-Asian rhetoric. For example, upon the COVID outbreak, some Asian countries from Thailand, South Korea, and Vietnam to Western countries like Italy put up signs forbidding Chinese people from entering restaurants and shops (Bauomy, 2020). Soon hate crimes targeting people of Asian descent went viral as illustrated above.

What we witness is that a virus linked to Chinese and Asian bodies is now further expanded to other racialized bodies, thus ‘racialized others’ are blamed for the outbreak and spread of the pandemic. For example, on February 28th, the leader of Italy’s La Lega party Matteo Salvini conflated Italy’s burgeoning outbreak with the recent arrival of a migrant boat from North Africa, and demanded an anti-immigration policy ‘iron-plating the borders’ although at that time Italy only had 288 reported cases of COVID-19, while the whole of the African continent had just a single case (Davis, 2020). In Nova Scotia, Canada, the Premier Stephen McNeil and Chief Medical Officer Robert Strang named the predominantly African Nova Scotian communities of Cherry Brook, Lake Loon, and the Prestons as locations of concern. They stated that “while we are using resources, doubling down on testing, and trying to keep people healthy, the reckless and selfish few in some of these communities are still having parties” (McSheffrey, 2020). These statements generated a storm of protest from the African Nova Scotian community. In several Chinese cities, many black people have faced serious racial discrimination and racism. For example, the majority Africans living in Guangzhou have been tested for COVID despite not showing any symptoms and black ethnic immigrants have been driven out of their houses and forced to self-quarantine (Qasim, 2020).

This *blaming racialized others* has been in full swing in the *blaming tactics* of national and international politics. Human Rights Watch (HRW, 2020) notes that government leaders and senior officials have explicitly and implicitly encouraged hate crimes, racism, or xenophobia by using anti-Chinese rhetoric, and several political parties in Italy, Spain, Greece, France, Germany, UK, and USA have also ‘latched onto the COVID-19 crisis to advance anti-immigrant, white supremacist, ultra-nationalist, anti-Semitic, and xenophobic conspiracy theories that demonize refugees, foreigners, prominent individuals and political leaders.’ They report two examples. In February, the governor of the Veneto region of Italy noted that Italy would be better than China in handling the virus because Italians have ‘culturally strong attention to hygiene, washing hands, taking showers, whereas we have all seen the Chinese eating mice alive.’ And in Brazil, the education minister ridiculed Chinese people in social media and suggested a paranoid conspiracy theory that COVID-19 was part of the Chinese government’s ‘plan for world domination.’

This blaming tactic is spreading in the world like the pandemic. India’s religious tensions between Hindu nationalists and Muslims has been well documented, but since the pandemic, Islamophobia has accelerated in India. A spree of Anti-Muslim attacks has followed India’s health ministry repeatedly blaming an Islamic seminary for spreading the coronavirus and hate speech has erupted across social media after government officials spoke of ‘corona jihad’ and ‘human bombs.’ In Canada, on May 13th Alberta’s Premier Jason Kenney criticized China’s handling of the COVID-19 outbreak, demanding ‘There must be some kind of a reckoning. There must be some accountability’ and the Chinese Consulate General in Calgary issued a statement criticizing Premier Kenney’s remark as ‘slander and stigma’ (Dryden, 2020). Again, noteworthy is not one conservative leader’s opinion but what it represents as a pervasive public rhetoric. According to the Angus Reid Institute (2020), 85 percent of Canadians perceive that the Chinese government has not been transparent about the pandemic in its own country.

In the midst of the blaming war between the Trump administration and the Chinese government, what is happening is a reification of *racist politics* for political agendas such as the imminent American presidential election. On May 29th President Trump announced unprecedented action against China: ‘They’ve ripped off the United States like no one has ever done before,’ he railed claiming that Beijing has ‘raided our factories’ and ‘gutted’ American industry, thus casting Beijing as the central foil he will run against in the remaining months of his re-election campaign. He even announced that the US would pull out of the World Health Organization claiming that China has total control over the WHO (Gaouette and Vazquez, 2020).

While there are complex political layers and histories between these two superpowers, this American presidential briefing clearly links the COVID pandemic to this polarising political agenda, laced with anti-China and anti-Asian rhetoric that is taken up to build his division between us and the ‘enemy,’ similar to the current pandemic being related to Islamophobia in India. Also, it is not difficult to see a parallel between the 2016 and the current election campaigns in the USA. In 2016 Trump took an anti-immigration position on the southern border of the US and now takes an anti-Asian position across the Pacific Ocean, while oppressing Latina and Asian – racialized bodies as the enemies that ‘ripped off,’ ‘raid’ and ‘gutted’ Americans. Similar to the fantasized image – Making America Great Again (MAGA) in 2016, the attack on Asian bodies during COVID-19 appears as merely another tactic to win out votes and to pave into extreme right-wing nationalism.

Blaming Asians/racialized others for the pandemic is not a new phenomenon, but rather part of ongoing racist politics using fear tactics to create an imagined enemy which consolidates the phantasy of US nationalism (Lee and Bhuyan, 2019). A law professor at the University of Helsinki, Hirvoner (2017: 249) argues that “racism is an elemental part of nationalist identity politics.” Building on his political application of the psychoanalytic concepts of fear and anxiety, we can see how “xenophobic images” (e.g., virus in Asian and racialized bodies), “nationalist signifiers” (e.g., Say No to Immigrants) and “racist fantasies” (e.g., MAGA campaign of building a wall on the southern border) create “the vicious circles of fear and hate” (e.g., hate crimes and fear of ‘danger out there’ in ‘them’) that “gives justification for the nationalist identity politics that raises security as the hegemonic organizing principle” (e.g., Trump legitimizes his unprecedented measures against peace and the global effort to mitigate the pandemic by provoking China and withdrawing from WHO). Canadian social work scholars, Lee and Bhuyan (2019) theorize this fear politics attesting that right-wing governments around the globe deploy nationalist slogans to deflect from internal troubles. For example, four non-white Democratic Congress Representatives – Ocasio-Cortez, Tlaib, Omar, and Pressley – point out that the fear-motivated racism and nationalism in Trump’s ‘xenophobic bigoted remarks’ deflects attention from the problems of affordable healthcare, student debt, and environmental pollution (Miller et al., 2019).

## Resisting the politics of the pandemic and racism

How should we address these fear-based politics of racism and nationalism amidst the pandemic? Hirvoner (2017:253) points out, “it is not enough to bring forth the factual evidence that would convince people that these fears are baseless and that the threats are exaggerated, or patiently deconstruct those hierarchical binary oppositions that form the driving force of the nationalist identity politics.” Rather, critical scholars strongly urge that it takes “emancipatory events that confront the racist and nationalist fantasy” (p. 249) – Indeed, social actions and calling out racism during the pandemic! Considering the current era of post-truth politics, we agree with these scholars that getting into a fight for facts and the patient deconstruction of politics is not enough, and there is a demand emancipatory resistance against the racist and nationalist politics which have blazed during the pandemic. We see the critical reflection QSW calls (Staller, 2020) for as such a form of resistance which is echoed by feminist scholars, Laura Brown and Susan Strega who highlight ‘research as resistance’ (2015).

As the emancipatory actions to fight against the racism during the pandemic, United nations (UN) Secretary-General Antonio Guterres urges governments to ‘act now to strengthen the immunity of our societies against the virus of hate’ and Asia Advisory Director John Sifton at HRW notes that ‘governments should act to expand public outreach, promote tolerance, and counter hate speech while aggressively investigating and prosecuting hate crimes.’ For example, in British Columbia (BC) Canada, the government has addressed the hate crime spike this spring. On May 16th Anne Kang, the Minister of Citizens’ Services and responsible for multiculturalism issued a statement in ten different languages to urge the end of hate crime, encouraging reporting of such incidents, and soliciting anti-racism proposals (Province of BC, 2020). On May 17th BC Premier John Horgan released a similar statement: ‘Racism is also a virus’ and he urged that ‘we must all stand together to call out racism and discrimination when we see it’ (Shepert, 2020). We call out for similar actions from all social workers to bear witness to identify, name, and resist the racism/pandemic in individuals, communities, and State policies while enduring the new norm of the global health crisis.

Albert Camus’ famous novel, *La Peste* (The Plague, 1947) has enjoyed a revival in this global pandemic era, in which the novel’s hero narrator Bernard Rieux, a physician, takes quiet steady moral actions amid his city’s devastation. Camus calls the plague the ‘virus Fascism without end’, something we cannot escape from and he shows that characteristic as human. Camus’ outlook certainly was drawn from his experiences during the Nazi occupation of Paris. Like literature where there are multiple interpretations of experience, we reflect on the Plague/COVID-19 as the allegory of the ‘virus of Fascism’/racist politics. Seven decades later, Sonia Shah (2020), the author of a recent book on the pandemic, describes a disturbing pattern of racism around the globe as the ‘shadowy pandemic’: ‘Even as public health experts race to contain the outbreak, a potentially more fearsome and shadowy pandemic – aimed at uninfected people unjustly fingered as potential carriers – grows.’ UN human rights chief Michelle Bachelet calls racism and xenophobia ‘contagious killers’, like COVID-19 (UN News, 2020). Indeed, the plague *is* human and with the coronavirus there comes another even more devastating pandemic, racism. An important message is communicated in the ending of *The Plague*: Rieux concludes that ‘those who knew now that if there is one thing one can always yearn for, and sometimes attain, it is human love’ (Vulliamy, 2015). In *Annals of Culture* on March 30th Issue, Lepore (2020) commented that ‘In the literature of pestilence, the greatest threat isn’t the loss of human life but the loss of what makes us human.’

In closing, we would like to invite readers to pause and reflect with us on the words of Chinese Canadian physician Margaret Chan who has been a global epidemic expert for the past 40 years and was the Director-General of WHO (2007-2017). While addressing 2009 H1N1 pandemic, she declared that “*After all, it really is all of humanity that is under threat during a pandemic”* (Harris and Altman, 2009). We echo her insight and further underline that the racism and the racist politics that have characterised this pandemic threaten all of humanity and all emancipatory actions such as noted above are helping what makes us human.
